# Carbohydrates Profile, Polyphenols Content and Antioxidative Properties of Beer Worts Produced with Different Dark Malts Varieties or Roasted Barley Grains

**DOI:** 10.3390/molecules25173882

**Published:** 2020-08-26

**Authors:** Justyna Gąsior, Joanna Kawa-Rygielska, Alicja Z. Kucharska

**Affiliations:** 1 Department of Fermentation and Cereals Technology, Faculty of Biotechnology and Food Science, Wroclaw University of Environmental and Life Sciences, Chełmońskiego 37, 51-630 Wrocław, Poland; joanna.kawa-rygielska@upwr.edu.pl; 2 Department of Fruit, Vegetable and Plant Nutraceutical Technology, Faculty of Biotechnology and Food Science, Wroclaw University of Environmental and Life Sciences, Chełmońskiego 37, 51-630 Wrocław, Poland; alicja.kucharska@upwr.edu.pl

**Keywords:** antioxidants, polyphenols, melanoidins, wort, malt, specialty malt, dark malt, beer, barley, roasting

## Abstract

The aim of this study was to assess the possibility of shaping properties of beers at the stage of brewing wort production with the use of various types of special malts (chocolate pale, chocolate dark, wheat chocolate, brown barley) and roasted barley grains. The carbohydrate profile, polyphenols content, antioxidant capacity, 5-hydroxymethylfurfural content, and the browning index level were analyzed. Statistical analysis showed significant differences in the values of the examined features between the samples. The sugars whose content was most affected by the addition of special malts were maltose and dextrins. The polyphenol content in worts with 10% of additive was 176.02–397.03 mg GAE/L, ferric reducing antioxidant power (FRAP) 1.32–2.07 mmol TE/L, and capacity to reduction radical generated from 2,2′-Azino-bis(3-ethylbenzothiazoline-6-sulfonic acid) diammonium salt (ABTS^•+^) 1.46–2.70 mmol TE/L. Wort with 40% dark malt showed the highest content of polyphenolic compounds and antioxidant activity (FRAP and ABTS^•+^). The HMF content and the browning index value were higher for wort with the addition of darker-colored malts (EBC) and increased with increasing dark malt dose.

## 1. Introduction

Composition of brewing wort is a crucial factor which forms quality of beer. The main raw materials used in the production of beer are malt and hops, which are also the main source of polyphenols. It is estimated that 80% of them come from malt and 20% from hops [1]. Polyphenols, as a part of a diet, contribute to maintaining the balance between the quantity of produced and deactivated free radicals, preventing oxidizing stress that causes numerous diseases such as obesity, diabetes type II, hypertension and atherosclerosis [2,3,4].

There are different types of malts used in brewery: base malts (Pilsner malt), malts made from non-standard (other than barley) grains (wheat, rye, oat, triticale, sorghum, maize etc.) and specialty malts (caramel, chocolate, roasted, melanoidin) [5,6]. Malts can be also produced from unusual materials such as leguminous plants seeds [7,8]. One of the quality indicators differentiating malts is their color described in EBC units (European Brewery Convention). The color range of malts ranges from 5 to 1600 EBC [6]. Dark malts are received through the process of roasting sprouted, not dried grains or roasting pale malt (previously dried). The malt houses offer a wide range of dark malts of varying color intensity. Examples include caramel malt (30–600 EBC), pale chocolate malt (350–450 EBC), dark chocolate malt (800–1000 EBC), melanoidin (1300–1500 EBC), roasted barley (900–1200 EBC) and others [9]. As a result of heat treatment they lose their enzymatic activity and therefore they are traditionally used only in small amounts (usually about 5%) compared to pale malts, which are enzymatically active [10]. Intendent use of specialty malts is colorization and production of characteristic flavors and aromas. Coghe et al. [11] indicated in a sensory evaluation that the intensity of aromas defined as roasted and bitter increases with the intensity of the wort color, while the perception of sweet taste and husky flavor decrease. The use of dark malts also affects the profile of flavor compounds, which are products of yeast metabolism in the fermentation process.

Malt is mainly produced from barley grains. Technological processes influence antioxidative activity of the final product. The grain in the malt house is subjected to soaking, germination, kilning and roasting in order to obtain malt with the desired characteristics [12]. During malting, numerous changes, resulting from the activity of enzymes, occur in barley grains. The main group of polyphenols in barley grain as well as in malt are flavon-3-ols, whereas the main phenolic compounds are (+)-catechin and ferulic acid. Components that cannot be found in barley grains, but are present in barley malt are (−)-epicatechin and sinapic acid. As a result of enzymatic transformations during malting, a change in polyphenol composition occurs in the grains. Among the compounds whose content is reduced we can distinguish catechin, prodelphinidin B3, procyanidin B3 and ferulic acid [13].

The technological process of beer production can also result in a phenolic profile change. In the final product the following phenolic compounds such as simple phenolic, derivatives of benzoic and cinnamic acids, coumarins, flavan-3-ols, proanthocyanidins, chalcones, flavanones, flavons, flavonols, α-acids and iso-α-acids can be distinguished [14]. The content of individual compounds, as well as the total content of polyphenols in beer, changes in the subsequent stages of production. Their final content in beer depends on the selection of raw material and wort production technology [15]. Melanoidins formed as a result of heat treatment of the malt made from reductive sugars and amino acids or proteins contribute to the forming of the antioxidative activity of worts and the final beers [16,17,18]. Colored macromolecules formed in the final stage of the Maillard reaction are subjected to a number of studies consequential to their interesting biological properties and unknown chemical structure [19]. Melanoidins positively affect antioxidative abilities of food products, as well as storage stability. The mechanism of their antioxidative activity relies on the ability to break chain reactions of radicals, chelation of metals, H_2_O_2_ reduction and scavenging of free radicals [16,17]. Melanoidins, apart from their antioxidative properties, also demonstrate antiviral and antimutagenic activity, and the ability to reduce cholesterol levels and stimulate growth of intestinal bacteria. However, some of the compounds, formed during early stages of the Maillard reaction process, are considered carcinogenic and mutagenic. Therefore, it is difficult to explicitly decide which of the activities are dominant [20,21]. One of the compounds formed during the heating of the malt is 5-hydroxymethylfurfural (HMF), which is also formed during thermal treatment of food, e.g., malt, dried fruit, fruit juices, coffee, bread or vinegar. Toxic effects of HMF consumption have been proved after receiving a dose of 75 mg/kg of body weight [22].

Research on antioxidant compounds which are natural food components is an important element of research on the prolongation of food products’ shelf life without the use of artificial preservatives [6]. The antioxidant activity of various types of beers and main raw materials used for their production has been repeatedly described in the literature. The influence of the addition of various malts, hops, fruits or fruit juices and their application on the physicochemical parameters and the sensory profile of beer have also been studied [23,24,25,26,27,28]. Additionally, it has been shown that dark beers possess a considerably higher content of polyphenols and antioxidative activity than pale beers [29,30,31]. This work focused on the analysis of the formation of the properties of beer through modifications at the primary stage of production—obtaining of the beer wort. Numerous publications present research that concerns the final product, but overlooks extremely important early stages of beer production process. The content of wort affects final beer quality and that is why it is necessary to focus on the properties of this intermediate product. The analysis of HMF content and the browning index along with the influence of these features on antioxidant activity is also interesting.

The aim of this study was to assess the possibility of shaping the properties of beers at the stage of brewing wort production with the use of various types of special malts (chocolate pale, chocolate dark, wheat chocolate, brown barley) and roasted barley grains. The purpose was achieved by the means of analysis of the carbohydrate profiles, total phenolic compounds content, antioxidative capacity, the level of browning and HMF content in worts produced with the use of specialty malts.

## 2. Results

### 2.1. Carbohydrates Profile

The analysis of the content of maltose, maltotriose, dextrins and glucose was carried out on wort prepared with the addition of 10% of selected special malts (light chocolate, dark chocolate or chocolate wheat, brown barley) or roasted barley grain (series I) and tests with 20–40% dark chocolate malt (series II). In each case, the remaining part of the malt charge was Pilsen malt. The control sample (P) was a wort made of 100% Pilsen malt. The results are shown in Table 1.

The sugars in series I were mainly maltose (51.8–55.71%), followed by dextrins (24.91–28.90%), maltotriose (13.9–14.64%) and glucose (0.75–6.80%). The statistical analysis showed significant differences in the content of the tested carbohydrates between the samples with the addition of special malts and the control sample, and between the samples with different types of special malts. The maltose content ranged from 34.74 ± 0.02 g/L in wort with the addition of wheat chocolate malt (PC) to 51.69 ± 0.33 g/L in wort with addition of roasted barley (JP). There are no statistically significant differences between the maltose content in the JP test and the control sample (P) (51.59 ± 0.01 g/L). These variants contained the most maltose among the worts in series I. The dextrin content ranged from 18.03 ± 0.30 g/L (PC) to 28.43 ± 0.08 g/L (JP). The results higher than the control sample were shown by the tests performed on the samples made with the addition of roasted barley grains (JP), brown barley (JB) and dark chocolate malt (CC), while the lower results were obtained for the samples with light chocolate malt (CJ) and chocolate wheat malt (PC). The amount of maltotriose in the trials ranged from 9.13 ± 0.01 (PC) to 13.87 ± 0.08 g/L (JP). Despite statistically significant differences, the amount of maltotriose clearly differed from the control sample only in the PC sample. The control sample contained 5.65 ± 0.19 g/L glucose. The JB showed higher glucose content. Statistical analysis showed no significant differences between the glucose content in the control sample and in the JP and CC samples.

In the series II worts, the highest proportion of sugars was maltose (43.85–58.07%), followed by dextrins (26.44–44.23%), maltotriose (11.92–15.27%) and glucose (up to 6.36%). Statistical analysis showed significant differences in the content of maltose, dextrins and maltotriose between the control sample and the series II variants. The control wort (P) contained the most glucose, maltotriose and maltose among the CC20-CC40 variants (wort with addition 20–40% of dark chocolate malt). With the increase in the share of dark chocolate malt, the content of dextrins not fermented by brewer’s yeast increased, while the content of maltose and maltotriose decreased. The highest content of maltose and maltotriose was found in the control wort (P), while the lowest was in the wort with the highest addition of dark malt (39.50 ± 0.06 g/L maltose and 10.74 ± 0.00 g/L maltotriose). The dextrin content ranged from 23.49 0.04 g/L to 39.85 0.16 g/L. All series II samples had a higher dextrin content than the control sample (P). No glucose was found in the CC20 and CC40. In the remaining samples, its content ranged from 2.80 ± 0.40 (for CC25) to the value close to the control sample, 5.60 ± 0.08 g/L (for CC35).

**Figure 1:**
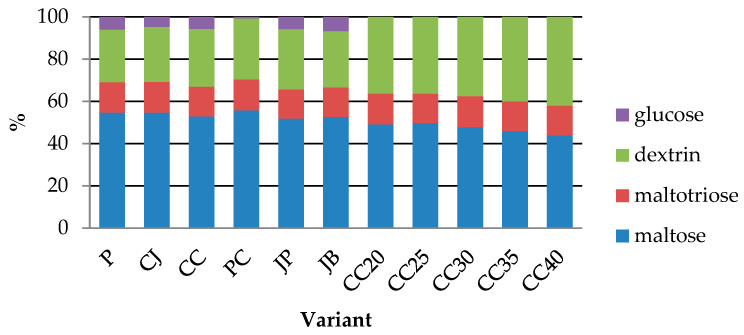
The share of individual sugars in the carbohydrate profile of the wort produced.

Figure 1 shows the shares of individual tested sugars in the carbohydrate profile of the control wort (P), worts in series I (CJ, CC, PC, JP, JB) and series II (CC20, CC25, CC30, CC35, CC40). In the control sample (P), maltose constituted 58.07% of the tested sugars, dextrins 26.44%, maltotriose 15.27%, and glucose 6.36%. Series I wort was characterized by a higher than control content of dextrins (26.06–28.9%), and a similar percentage of maltotriose (13.90–14.64%). The addition of special malts in the dose of 10% had a slight effect on the maltose content in the wort. Its share ranged from 51.80–55.71%. A significantly lower share of glucose content was characteristic for the PC wort (0.75%). On the other hand, the JB wort had a higher glucose share (6.8%). In the remaining wort, the share of glucose in the sugar profile was 4.72–5.81%.

Series II worts were characterized by a lower than control share of maltose (43.8–48.75%) and maltotriose (11.92–13.51%), and a higher share of dextrins (36.88–44.23%). The share of dextrins in the total sugars of the wort increased with the increase in the share of dark chocolate malt in the malt charge. The opposite trend was observed for the content of maltotriose and maltose. Their content decreased with increasing dose of special malt. The CC30 and CC35 samples had the highest share of glucose, comparable to the control sample, in the carbohydrate profile.

**Figure 2:**
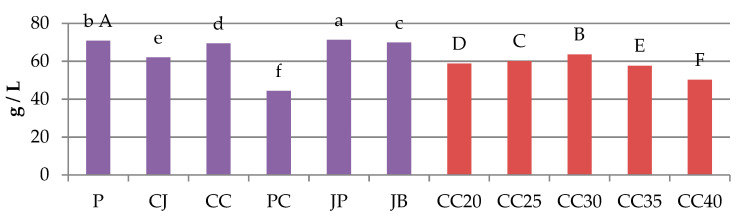
Content of fermentable sugars (glucose, maltose, maltotriose) in wort. Values are expressed as sums of the means of 3 replications (*n* = 3) for the fermentable sugars tested (glucose, maltose, maltotriose). Mean values with different letters (a, b, c, d, e, f or A, B, C, D, E, F) are statistically different (*p*-value < 0.05).

Figure 2 shows the content of fermentable sugars (maltose, maltotriose and glucose) in the control wort (P), series I trials (CJ, CC, PC, JP, JB) and series II trials (CC20, CC25, CC30, CC35, CC40. In the series I trials, JP had the highest content of sugars fermented with brewer’s yeast. These results were close to the P, CC, and JB samples. It confirms that the addition of 10% special malts does not cause major changes in the sugar profile of the wort. The lowest content of fermentable carbohydrates was characteristic for the PC sample. The use of another variety of grain led to significant changes in the composition of the brewing wort. The content slightly varied between the tests of CC20, CC25 and CC30. With a higher proportion of special malt in the powder, the content of fermentable sugars began to drop significantly.

### 2.2. Concentration of Total Polyphenols and Antioxidative Activity

Wort made with different types of dark malt and roasted barley grain (series I) and wort produced with different proportions of dark chocolate malt (series II) were tested for the content of phenolic compounds and antioxidant activity. The results are shown in Table 2.

CJ, CC, PC, and JP wort contained significantly more phenolic compounds than the control sample. The JP variant was characterized by a similar phenols content to the control sample. The worts CC (404.38 ± 5.98 mg GAE/L) and JP (397.03 ± 7.63 mg GAE/L) had the highest total polyphenol content among the series I samples. The remaining worts showed a lower phenolic content. The worts were also analyzed for the ability to reduce iron ions (FRAP) and the antioxidant capacity (by the means of ABTS^•+^ assay). Similarly to the content of total polyphenols, the CC wort (2.07 ± 0.03 mmol TE/L) was characterized by the highest FRAP value. The wort with the lowest ability to reduce iron ions was JB (1.32 ± 0.02 mmol TE/L). All the samples of series I showed a FRAP ability higher than the control. In the analysis of capacity to reduction of ABTS^•+^ the JB wort showed the highest result (2.70 ± 0.40 mmol TE/L). The CC wort was also characterized by a high activity of scavenging ABTS^•+^ radicals (2.17 ± 0.17 mmol TE/L).

The wort produced with different proportions of dark chocolate malt (series II) were also analyzed. The control sample was characterized by the lowest total phenol content (192.58 ± 18.66 mg GAE/L) and the lowest antioxidant activity measured with both methods (FRAP, ABTS^•+^). Along with an increase in the share of CC malt in the range of 20–40%, an increase in the content of polyphenols, the ability to reduce iron ions, and the ABTS^•+^ antioxidant capacity were observed. The CC40 wort contained the most polyphenols (922.32 ± 6.96 mg GAE/L). The content of polyphenols increased in proportion to the applied dose of dark malt. As a result, the lowest polyphenol content among worts with special malt was characterized by the CC20 wort (593.82 ± 26.23 mg GAE/L). Similar trends were observed for the antioxidant activity (ABTS^•+^ and FRAP).

### 2.3. Browning Index and Concentrations of 5-Hydroxymethylfurfural (HMF)

The browning index analysis of samples using the spectrophotometric method of measuring absorbance at 420 nm is the simplest method of assessing the content of colored compounds. The content of HMF and the browning index are presented in the Table 3.

Among the wort from series I, the JP variant (5.57 ± 0.03 AU) was characterized by the highest value of the browning index. The parameter value decreased in the following sequence: CC > PC > CJ > JB. The control sample had the lowest parameter value (0.62 ± 0.04 AU). In series II, along with the increase in the share of special malt, the level of browning index increased. The absorbance results ranged from 10.05 ± 0.07 AU to 17.99 ± 0.48 AU. Statistical analysis classified all the results into separate statistical groups. The HMF content increased with increasing heat treatment time or temperature. In the series I trials with the addition of various types of special malts, it was shown that the highest HMF content among the tested samples was shown by JP (24.31 ± 0.17 mg/L). The analysis results for series I trials decrease in the following sequence: CC > CJ > JB > PC. The lowest HMF content was recorded for the control sample (0.66 ± 0.00 mg/L). The highest HMF content was shown by the CC40 variant, as expected. Another test with high HMF content was the CC35 test (75.61 ± 0.43 mg/L). The differences between the remaining trials were smaller, but statistically significant. They contained from 47.62 ± 0.54 to 51.81 ± 0.54 mg/L of HMF.

The color of the wort expressed in absorbance units differed significantly between the samples, indicating the highest value for the JP (5.57 ± 0.03 AU), and the lowest for the control sample P (0.62 ± 0.04 AU). The color of the wort defined as the browning level was related to the color of the malt used in the production of the wort or the dose of dark malt used. With the increase in the malt color intensity expressed in EBC units, the absorbance value in the browning analysis increased.

## 3. Discussion

### 3.1. Carbohydrates Profile

According to results of Coghe et al. [32] the addition of 10% of special malts did not cause such significant changes in the carbohydrate profile of the wort, although it had an adverse effect on the subsequent ethanol fermentation process, causing a decrease in the degree of attenuation and ethanol content in beers. Maltose and maltotriose were the main sugars in the brewing wort in all trials of our experiment. This is consistent with the results of Briggs et al. [33]. Coghe et al. [32] also showed that in a wort made of 100% Pilsen malt, the amount of maltose is about 70% of the main fermentable sugars, about 18% is maltotriose, and 12% is glucose. The discrepancy in our results may be due to the use of different parameters of the mashing process or malt with different properties. According to Briggs et al. [33], maltose is usually between 50 and 60% of the sugars in the wort. In our research, only the wort prepared with 40% of special malt has a lower maltose content than 50%. The addition of dark malts in the wort production reduces the content of fermentable sugars [33]. Coghe et al. [32] analyzed wort with the addition of 50% special malts (7–900 EBC) and showed that the maltose content decreases as the color intensity of the malt increases in the studied range. In the worts prepared with the darkest tested malts, a decrease in the content of glucose, fructose, and maltotriose was also observed in comparison with the control wort from Pilsen malt [32]. Some of the sugars are used in non-enzymatic browning reactions during the thermal treatment of cereal grains. This is the main reason for reducing the content of fermentable sugars during heat treatment [34].

The differences in the sugar content between JP and other samples result not only from the difference in their color. Contrary to the other tested samples, these grains are not malted before the roasting process. The malting process leads to an increase in the content of maltose, sucrose and glucose, while slightly modifying the content of maltotriose and fructose [35]. Kilning and roasting, on the other hand, decrease the content of fermentable sugars. The final sugar content in the tested products is modified by a number of factors, ranging from the chemical composition, the treatment method used, grain germination, and kilning and roasting parameters.

The carbohydrate profile of brewing wort is closely related to the type of malt used in their production. The tested raw materials differ in terms of production technology. JP is roasted, unmalted barley grains, while the remaining grain samples (CC, CJ, PC, JB) are grains which were subjected to the processes of soaking, germination, kilning and roasting, so they are products with a very different chemical composition. Wheat grain may have a higher maltose content and a lower glucose content than barley, therefore the addition of wheat malt could cause changes in the composition of these sugars in the wort [36]. The glucose content of the roasted unmalted grain sample (JP) is significantly higher than in CC, CJ, and PC. According to Vinje et al. [37] in the process of grain germination, the content of maltose, maltotriose, and glucose increases in the grain due to the activity of enzymes. This suggests that the malts used should have a higher content of these sugars than unmalted grains.

According to Briggs et al. [33], the addition of special malts reduces the obtained wort extract, which consists of the content of sugars such as glucose, fructose, sucrose, maltose, and maltotriose [31]. In the case of the production of brewing wort, the toxic effect of melanoidins on yeast metabolism is important. Coghe et al. [31] tested worts with the addition (5–50%) of caramel malt (300 EBC) and Pilsen malt. The addition of dark malts had not significantly affected the apparent extract of the wort, while the samples were clearly differentiated in terms of the real extract, and thus also the degree of attenuation and the final ethanol content in the beer. This may indicate that the content of sugars in the darker worts may be less susceptible to fermentation or that the substances produced as a result of the Maillard reaction have an inhibitory effect on yeast metabolism [32]. According to our results, dextrin concentration increases with the content of the dark malts used. Dextrins do not undergo ethanol fermentation using the classic strains of *Saccharomyces cerevisiae* brewer’s yeast, therefore their amount in the wort is similar to that in the beer [38].

### 3.2. Concentration of Total Polyphenols and Antioxidative Activity

Polyphenolic compounds are responsible for antioxidant activity and storage stability, but can also cause haze in beer [1]. The ability to scavenge free radicals is also demonstrated by compounds formed in the Maillard reactions that are formed during the thermal treatment of malt and wort. Barley grains and barley malts used in brewing are dried and roasted. As a result, a number of products are obtained, differing in sensory characteristics as well as physicochemical properties. During roasting, the main chemical reactions are the Maillard reactions. These are a series of changes initiated by the reaction between reducing sugars and amino groups, leading to the formation of compounds responsible for the color and flavor of heat-treated products. The non-enzymatic browning reactions can be divided into several stages. In the first of them, the early phase reaction products are created. They are mainly products of the Amadori rearrangement. The second step involves the formation of intermediates such as: HMF, Strecker aldehydes and pyrazines. The last, third stage is the formation of the melanoidins, which are reaction end products [39]. Melanoidins are a group of compounds with a very diverse structure. They differ in chemical properties depending on the origin. Melanoidins give beer its color and provide it with antioxidant activity [40]. The antioxidant activity of the Maillard reaction products is confirmed by studies on the antioxidant properties of coffee. As the degree of roasting of coffee beans increases, some polyphenolic compounds are lost, however this is not correlated with a decrease in the antioxidant activity of coffee infusions. In place of these compounds, new chemicals are created, which also act as antioxidants [41]. Similar tendencies can be observed in the case of the wort tested in our experiment.

Polyphenolic compounds undergo chemical changes under the conditions of the technological process of malt and wort production. This happens during the thermal processing of the grains. For some polyphenols (e.g., quercetin), heat treatment is associated with an increase in antioxidant activity. Their decomposition products are more powerful antioxidants than the original compounds [42]. This proves the influence of the compound’s structure on its antioxidant properties. The antioxidant activity is enhanced by the presence of certain chemical groups in the structure of phenols and the degree of polymerization of the compound [43]. Woffenden et al. [44] showed a significant increase in the content of catechin and ferulic acid during the malt kilning process. An increase in the antioxidant capacity ABTS^•+^ and the ability to reduce iron ions (FRAP) of malt during the process was also observed. Polyphenolic compounds are extracted during the production of the wort and build its antioxidant activity. Fogarasi et al. [45] demonstrated that wheat malt is characterized by a lower content of phenolic compounds than barley malt. The differences in the content of polyphenolic compounds in the tested samples may be the result of the addition of raw materials of different varieties (barley, wheat), different colors (3–1300 EBC), and different production technologies (malting process parameters).

On the other hand, it was shown that the malt kilning process led to a reduction in ferulic acid content. Melanoidins may have the ability to cross-link simple phenolic compounds in their structure, leading to a decrease in the content of phenolic compounds. High temperatures may also affect the activity of enzymes (e.g., ferulic acid esterase) responsible for the release of phenolic compounds from the cell walls [31,46]. This explains the decrease in the ferulic acid content of the malt compared to the corresponding cereal grain. In the wort obtained in the experiment, the antioxidant activity increased with the increase in the dose of dark malts and was higher for wort obtained from darker-colored malts. Dark special malts are made through a high temperature treatment. The increase in the content of phenolic compounds during the production of malt occurs at the stage of germination and kilning. Grain soaking is the cause of the loss of some of these bioactive compounds [31,45]. Roasted barley grains are not subjected to soaking and germination, therefore their polyphenol content may differ from malts.

Despite the chemical changes that take place in the wort during the fermentation and aging process, when obtaining a wort with a higher content of polyphenolic compounds and antioxidant activity we can expect a higher content of polyphenolic compounds in the finished product. Due to this, it is worth striving to achieve a high dose of these bioactive compounds already at the stage of wort production.

### 3.3. Browning Index and Concentrations of 5-Hydroxymethylfurfural (HMF)

The browning index increased with the darkening of the wort color. This is consistent with the results [47] and is similar to the content of HMF. The HMF content is one of the indicators of the non-enzymatic browning reaction, as it is formed as an intermediate product of the reaction [20]. There are two main mechanisms of its formation in food. The first is the transformation of 3-deoxyglucosan during the Maillard reaction, the second is the dehydration of sugars under acidic conditions (caramelization) [48]. HMF is found in a wide variety of foods. In beer, its content ranges from 0.2–9.2 mg/kg. It can also be found in other alcohols: wine (0.1 mg/L), whiskey (8.6 mg/L), white spirit (2.2 mg/L), brandy (22 mg/L), rum (8.4 mg/L), and others [49]. Among other food products, it can also be found in honey (0.34–58.8 mg/kg), cookies (1.75–35.21 mg/kg), bread (2.2–22 mg/kg), dried fruit (25–2900 mg/kg), coffee beans (100–2186 mg/kg), instant coffee (91.3–4100 mg/kg), fruit juices (2–22 mg/kg), chocolate (42–99 mg/kg), or breakfast cereals (12–47 mg/kg) [50,51]. In brewing malt its content varies widely and ranges from 100–6300 mg/kg, while in the grain of barley it is 100–1200 mg/kg [52,53]. Compared to other food products, the HMF content in beer is relatively low. The low content of HMF in the beer compared to the values obtained in the wort results from the potential of *Saccharomyces cerevisiae* brewer’s yeast to reduce this compound during the ethanol fermentation process. HMF has been shown to be converted by yeast into hydroxymethyl furfuryl alcohol with high efficiency (79–84%). Moreover, it has been shown that the degradation of HMF is carried out by yeast preferentially, faster than ethanol fermentation [53]. The HMF content in commercial beers in different styles has been analyzed. It was shown that blond beers contain HMF in range of 2.42–5.80 mg/L, amber beers 5.92–7.44 mg/L, and dark beers 6.29–7.52 mg/L. Moreover, there is no clear difference between top fermented (ale) and bottom fermented (lager) beers in the content of HMF [54]. Coffee (50.43%) and white bread (31.8%) have the highest share in the daily consumption of HMF by humans. Beer, on the other hand, influences the daily dose of HMF by 2.86% [55]. Both positive and negative effects of HMF consumption by humans have been reported [51].

### 3.4. Correlation and Linear Regression Analysis

**Figure 3:**
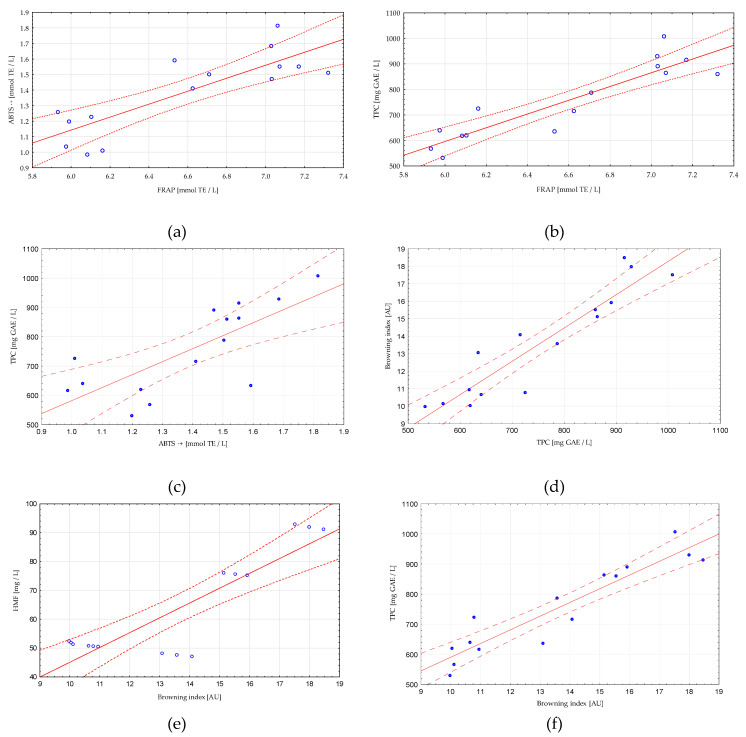
Scatter graphs for selected variables. Relationship between: (**a**) FRAP and ABTS^•+^ assays, (**b**) FRAP and TPC (content of polyphenols) assays, (**c**) ABTS^•+^ and TPC assays, (**d**) TPC and browning index, (**e**) HMF and browning index, (**f**) browning index and TPC.

Multiple linear regression analysis was used to investigate the relationship between tested parameters and the wort composition. The results are presented in Table 4 and Figure 3, showing a number of relationships between the content of sugars, polyphenols and the antioxidant activity of worts produced with the use of 20–40% dark chocolate malt in the raw material composition.

A strong positive correlation was observed between the content of polyphenols (TPC) and the level of browning index expressed in absorbance units (r = 0.93), the ability to reduce iron ions (FRAP) (r = 0.91), antioxidant capacity ABTS^•+^ (r = 0.75), the content of HMF (r = 0.87) and dextrins (r = 0.82). The influence of polyphenolic compounds on the antioxidant activity has already been demonstrated many times [26,27,28,29]. The analysis showed significant correlation between the results of the antioxidant capacity test with the ABTS^•+^ and FRAP methods. Tests for the analysis of antioxidant activity are based on various mechanisms. In both the FRAP and ABTS^•+^ analyses, we investigated the ability to transfer a single electron from a compound with anti-oxidative abilities to an oxidant. These methods belong to the group of methods based on the SET (single electron transfer) mechanism. Nevertheless, they do not have to show conclusive results. An example is the study by Müller et al. [56], where the authors compared the antioxidant capacity with the use of various analytical methods, obtaining divergent results. Therefore, it is suggested to use several research methods simultaneously. It has also been shown that one of the main polyphenols of malt wort, i.e., ferulic acid and catechin, showed a higher value for ABTS^•+^ than for FRAP (in both methods the results were presented in terms of the Trolox equivalent). Moreover, the ABTS^•+^/FRAP ratio for the plant extracts analyzed by the authors varies widely, from 0.7 to 3.3 [57].

The content of polyphenolic compounds is also significantly negatively correlated with the content of maltose (r = −0.91) and maltotriose (r = −0.91). The amount of sugars is also related to the browning index level (r = −0.88 for maltotriose, r = −0.91 for maltose, r = 0.94 for dextrin). Similar trends occur in the case of HMF content (r = −0.88 for maltotriose, r = −0.98 for maltose, r = 0.80 for dextrin). Woo et al. [58] analyzed the formation of HMF during the thermal processing of glucose and maltose. It has been shown that thermal processing of maltose produces slightly less HMF compared to processing glucose. The content of dextrins in the wort is strongly related to the dose of dark malt and its concentration with its increase, which was demonstrated in this study.

The content of HMF and the browning indexes are indicators of the presence of Maillard reaction compounds derived from heat-treated malt. It was shown that the value of both these features was strongly correlated with the results of measuring the content of polyphenolic compounds by the Folin–Ciocalteu method and the antioxidant activity (FRAP and ABTS^•+^). Bastola et al. [59] analyzed the results of the polyphenol content (F–C) for interference with the mixture of phenolic compounds, sugars (including glucose, which belongs to the reducing sugars), and HMF. It was shown that the content of glucose and HMF did not affect the level of phenols determined by the F–C method. The analysis showed a strong correlation between the content of maltotriose and maltose and the level of browning. A correlation was not observed with glucose. α-dicarbonyl compounds formed in the advanced stage of the Maillard reaction have a key influence on the type and structure of high-molecular melanoidins responsible for the color formation. Studies on Maillard reaction kinetics prove that oligomeric carbohydrates have a greater influence on the intensity of the browning reaction, and this influence increases with the increase of the compound polymerization level. This is due to the lower number of carbonyl groups in the oligosaccharides [60].

The analysis of the correlation between the examined features allows for the demonstration of a number of dependencies that are introduced by the increase in the share of dark chocolate malt in the composition of the brewing wort. Conducting such an analysis is a valuable tool that allows for a better visualization and understanding of changes in physicochemical parameters of the wort related to the change in the composition of raw materials.

## 4. Materials and Methods

### 4.1. Raw Material

For the wort production we used: Pilsen malt 3–4.3 EBC (Viking Malt, Strzegom, Poland), pale chocolate malt 350–450 EBC (Viking Malt, Strzegom, Poland), roasted barley > 1000 EBC (Viking Malt, Strzegom), wheat chocolate malt 800–1000 EBC (Thomas Fawcett & Sons, Castleford, Great Britain), barley brown 175–200 EBC (Viking Malt, Strzegom, Poland), dark chocolate malt 1100–1300 EBC (Viking Malt, Strzegom, Poland).

### 4.2. Prepartation of the Congress Worts

**Figure 4:**
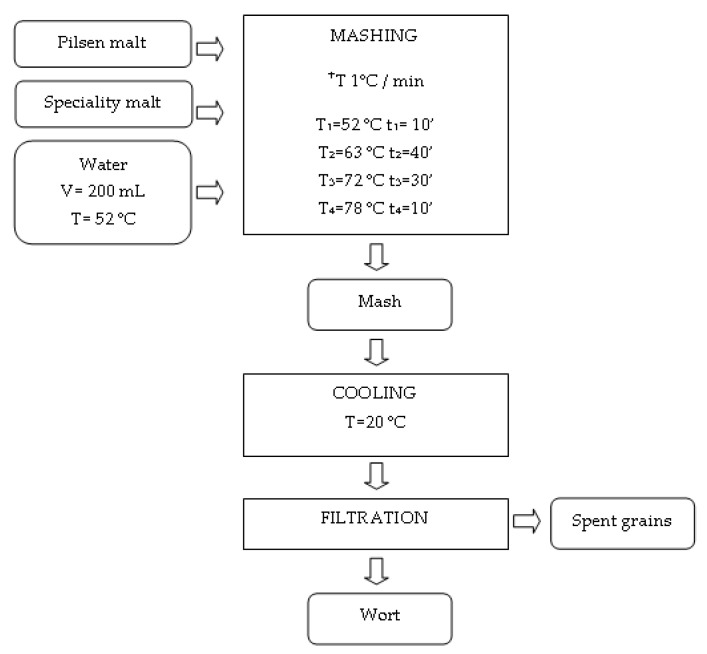
Technological process of producing wort.

Malts were ground with the mill for malts model VLB DLFU W20050 and mashed with the laboratory masher VLB type LB. A total of 12.50 g of malt blends was weighed in a cup and complemented with 200 mL distilled water (T = 52 °C). Cups were weighed and put in the masher along with the stirrer. The mash program was performed as follows: 52 °C (10 min), 63 °C (40 min), 72 °C (30 min) and 78 °C (10 min) (Figure 4).

After the mashing was completed, the iodine test was performed. Mashes were cooled down to 20 °C, filled up with water until primary mass was achieved and filtered through paper filters into an Erlenmeyer flask, while first returning 100 mL of the filtrate. The extract content in each sample was set for 12°Bx. Wort extract was studied using density meter 30PX (Columbus, OH, USA) in 20 °C. The obtained worts were centrifuged with laboratory centrifuge type MPW-351R in falcon containers with the capacity of 50 cm^3^ using 5000 rpm for 10 min.

**Figure 5:**
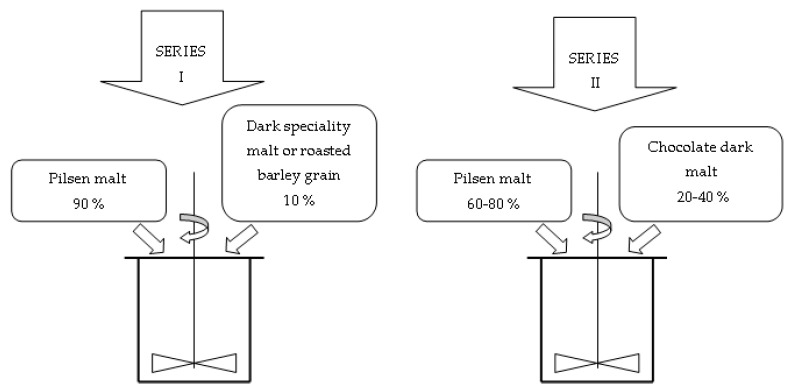
Wort production scheme.

Obtained samples were decanted. Two series of wort were obtained (Figure 5): series I–with the addition of 10% pale chocolate malt (CJ), dark chocolate malt (CC), wheat chocolate malt (PC), roasted barley (JP) or brown barley (JB), series II–with the addition of 20% (CC20), 25% (CC25), 30% (CC30), 35% (CC35) or 40% (CC40) chocolate malt dark and control wort made of 100% Pilsen malt (P).

### 4.3. Analytic Methods

#### 4.3.1. High-Performance Liquid Chromatography (HPLC) Analysis of Carbohydrate Profile

Carbohydrate profiles of worts were determined using high-duty methods of liquid chromatography (HPLC) [61]. Samples were centrifuged with laboratory centrifuge type MPW-351R (10 min, 5000 rpm) and subjected to 2-times dilution. Separation of the mixture was made using Rezex ROA Organic Acid H^+^ (300 × 7.8 mm) column made by Phenomenex. Respectively, a method of high-duty chromatography HPLC, using Shimadzu Prominence apparatus, was used to analyze concentration of glucose, maltose, maltotriose, dextrin and amount of produced glycerol and ethanol. A volume of 0.02 cm^3^ injection, celerity of the flow of eluent 0.6 cm^3^/min, temperature of separation 60 °C, solution H_2_SO_4_ 0.005 mol/dm^3^ as eluent and refractometric method of detection were used.

#### 4.3.2. High-Performance Liquid Chromatography (HPLC) Analysis of 5-Hydroxymethylfurfural (HMF) Content

HMF content was determined using high-performance liquid chromatography [62]. For the analysis we used a Dionex system (Thermo Fisher Scientific, Germering, Germany) equipped with diode detector UltiMate 3000, LPG-3400A pomp, EWPS-300SI autosampler and thermostated column TCC-3000SD. Cadenza Imtakt CD-C18 (75 × 4.6 mm, 5 μm) column was used. Moving phase was A (4.5% aq. formic acid, *v*/*v*) solvent and B (100% acetonitrile).

Following the procedure of elution we used: 0–1 min 5% B in A, 20 min 25% B in A, 21 min 100% B, 26 min 100% B, 27 min 5% B in A. Celerity of the flow was 1.0 mL/min, and the volume of injection 20 μL. Temperature of operating column was 30 °C. HMF was detected at 280 nm. Integration and quantification of the data was made using the software Chromeleon v.7.2.-Chromatography Data System (Thermo Scientific Dionex, Sunnyvale, CA, USA). Results were in mg/L of the wort.

#### 4.3.3. Total Polyphenols Content

Total content of polyphenol compounds in worts was determined using spectrophotometric Folin–Ciocalteu (F–C) method [63]. Samples of the worts with the volume of 0.1 mL along with 0.2 mL of F–C reagent were placed in the cups using automatic pipette. After 3 min each sample was completed with 1 mL 20% of the water solution of sodium carbonate (Na_2_CO_3_) and 2 mL of distilled water. After 1 h, prepared samples were analyzed using UV-2401 PC Shimadzu spectrophotometer with wave length 765 nm; distilled water was used as a blind sample. Results were presented as an average value from three repetitions. Calibration curve in the range of 0.30–9.00 mg GAE/L was used to read the results.

#### 4.3.4. Antioxidative Activity

##### Ability to Iron Ions Reduction (FRAP)

The principle of the FRAP method (ferric ion reducing antioxidant parameter) is the reduction of iron-2,4,6-tri(2-pyridyl)-1,3,5-thiazide [Fe(III)-TPTZ] to the ferric complex in the environment of low pH [64]. A total of 200 mL of FRAP reagent was made by combining 20 mL of a water solution containing 0.1018g of iron chloride (III) (FeCl_3_) with a solution of 0.0664g TPTZ in 20 mL 40 mmol solution of muriatic acid (HCl) using acetate buffor with pH 3.6.

Quantitative analysis was made using method of the external standard using iron (II) (2 × 10^−1^ mmol/L) sulfate (VI) as a referential standard. On this basis, a correlation curve between absorbency value and compound concentration was made. A total of 1 mL of beer dissolved in distilled water and 3 mL of FRAP reagent was mixed in cups. Results were presented in milimoles of Trolox per liter of the wort. Absorbance was determined using spectrophotometer UV-2401 PC Shimadzu. Determinations were performed in triplicate.

##### Ability to Cation Radical ABTS^•+^ Reduction

Antioxidative activity was determined using cation radical reduction method ABTS^•+^ [65]. Thus, 0.03 mL volume wort sample was mixed with the ABTS^•+^ solution with determined absorbance value (0.700). After 6 min, measurement was made using UV-2401 PC Shimadzu spectrophotometer with the wave length 734 nm. The measurement of each sample was conducted and repeated three times. Results were presented after calculation for mmol equivalent of Trolox for liter of the beer and as a percentage value (I%) of inhibition of oxidation reaction compared to the blind sample.

(1) $$I\%=\frac{A}{A_{0}}\times 100\%$$

I%—inhibition of oxidation reaction degree (%); A—absorbance value for the actual sample; A_0_—absorbance value for the reference sample.

#### 4.3.5. Browning Index

Browning index was estimated using absorbance measurement method at 420 nm [66]. The analysis was made using UV-Vis Evolution 300 spectrophotometer (ThermoScientific, Waltham, MA, USA). The results were presented as arbitrary absorbance units (AU).

#### 4.3.6. Statistical Analysis

Obtained data were analyzed using Statistica 13.5 (StatSoft, Tulsa, OK, USA). One-way variation analysis (ANOVA) on the statistical significance level 0.05 was performed. Significance of the differences between mean values was examined using Duncan test (*p* < 0.05). In order to study connections between examined characteristics, a Pearson correlation (*p* < 0.05) analysis was conducted. The results were presented as a correlation matrix and a regression diagram.

## 5. Conclusions

Our research confirms that the use of dark malts and roasted cereal grains in a 10% dose in the production of brewing wort influences antioxidant potential and the content of phenolic compounds of the worts, with slight changes in the carbohydrate profile. Among the tested worts, the wort with the addition of dark chocolate malt and roasted barley had a distinguishing polyphenolic content and antioxidant activity. Samples differed in terms of the tested parameters due to the type of roasted grain used. The wort with the addition of wheat malt was characterized by a lower sugar content than the other worts made of malts or barley grains. It has been shown that with increasing dose of dark chocolate malt, the share of dextrins not fermented by the brewer’s yeast increased, while the content of fermentable sugars decreased. In the study of wort containing 20–40% dark chocolate malt, it has been shown that a high addition of this malt resulted in an increase in the content of polyphenols and the antioxidant capacity as well as the content of HMF and browning index. Information about the factors influencing the antioxidant activity were obtained by correlation analysis. The ability to reduce iron ions (FRAP) and capacity for reduction of ABTS^•+^ is strongly positively correlated with the content of polyphenols, the level of browning index, and the content of dextrins, and negatively correlated with the content of maltotriose and maltose. This research allowed us to present the relationships between the tested properties of the brewed worts. The addition of dark malts can be used to increase the antioxidant activity of the wort, influencing the characteristics of the finished beer. The ability to scavenge free radicals may shape the biological activity of products and contribute to the maintenance of product quality during storage. An interesting direction for further research would be the study of the addition of other roasted raw materials, e.g., coffee beans or cocoa, as well as the use of untypical malts in beer production.

## Figures and Tables

**Table 1:** Carbohydrate profile of wort with the addition of special malts.

Series	Variant	Maltose	Dextrin	Maltotriose	Glucose
		g/L			
	**P**	51.59 ± 0.01 ^a,A^	23.49 ± 0.04 ^d,E^	13.57 ± 0.02 ^b,A^	5.65 ±0.19 ^b,c,A^
**I**	**CJ**	45.86 ± 0.40 ^c^	21.86 ± 0.40 ^e^	12.21 ± 0.10 ^e^	3.96 ± 0.15 ^d^
	**CC**	50.50 ± 0.04 ^b^	26.09 ± 0.22 ^b^	13.46 ± 0.01 ^c^	5.48 ± 0.14 ^c^
	**PC**	34.74 ± 0.02 ^c^	18.03 ± 0.30 ^f^	9.13 ± 0.01 ^f^	0.47 ± 0.06 ^e^
	**JP**	51.69 ± 0.33 ^a^	28.43 ± 0.08 ^a^	13.87 ± 0.08 ^a^	5.80 ± 0.02 ^b^
	**JB**	50.14 ± 0.06 ^b^	25.31 ± 0.23 ^c^	13.34 ± 0.00 ^d^	6.48 ± 0.04 ^a^
**II**	**CC20**	46.12 ± 0.18 ^B^	35.06 ± 0.17 ^C^	12.68 ± 0.10 ^B^	nd
	**CC25**	45.50 ± 0.23 ^C^	33.73 ± 0.32 ^D^	11.83 ± 0.12 ^D^	2.80 ± 0.40 ^C^
	**CC30**	45.71 ± 0.41 ^B,C^	37.22 ± 0.22 ^B^	12.05 ± 0.11 ^C^	4.18 ± 0.34 ^B^
	**CC35**	41.17 ± 0.22 ^D^	37.43 ± 0.22 ^B^	10.90 ± 0.06 ^E^	5.60 ± 0.08 ^A^
	**CC40**	39.50 ± 0.06 ^E^	39.85 ± 0.16 ^A^	10.74 ± 0.00 ^F^	nd

Abbreviations: nd—not detected; series I—wort with 10% addition of one of the following special malts: pale chocolate malt (CJ), dark chocolate malt (CC), wheat chocolate malt (PC), roasted barley (JP) or brown barley (JB); series II—wort with the addition of 20% (CC20), 25% (CC25), 30% (CC30), 35% (CC35) or 40% (CC40) of dark chocolate malt; control wort made of 100% Pilsen malt (P). Values are expressed as mean of 3 replications (*n* = 3) ± standard deviation. The mean values with different letters (a, b, c, d, e, f or A, B, C, D, E, F) in the same column and within one series are statistically different (*p*-value < 0.05).

**Table 2:** Characteristics of wort produced with selected dark malts and roasted barley grains in terms of polyphenol content and antioxidant activity expressed with FRAP (ferric reducing antioxidant power) and ABTS^•+^ (2,2′-Azino-bis(3-ethylbenzothiazoline-6-sulfonic acid) diammonium salt) methods.

Series	Variant	Total Polyphenol Content (F–C)	FRAP	ABTS^•+^	
		mg GAE/L	mmol TE/L	mmol TE/L	% Inhibition
	**P**	192.58 ± 8.66 ^d,F^	1.23 ± 0.01 ^f,D^	1.44 ± 0.09 ^c,B^	22.17 ± 0.87
**I**	**CJ**	252.84 ±12.81 ^c^	1.66 ± 0.03 ^c^	1.76 ± 0.17 ^c^	26.82 ± 1.70
	**CC**	404.38 ± 5.98 ^a^	2.07 ± 0.03 ^a^	2.17 ± 0.17 ^b^	32.78 ± 1.80
	**PC**	303.90 ± 7.83 ^b^	1.44 ± 0.01 ^d^	1.46 ± 0.11 ^c^	22.53 ± 1.13
	**JP**	397.03 ± 7.63 ^a^	1.90 ± 0.02 ^b^	2.70 ± 0.40 ^a^	40.33 ± 4.06
	**JB**	176.02 ± 0.59 ^d^	1.32 ± 0.02 ^e^	1.46 ± 0.05 ^c^	22.53 ± 0.51
**II**	**CC20**	593.82 ± 6.23 ^E^	6.01 ± 0.09 ^C^	1.23 ± 0.03 ^C^	19.19 ± 0.31
	**CC25**	628.79 ± 10.73 ^D^	6.10 ± 0.09 ^C^	1.01 ± 0.03 ^D^	16.06 ± 0.26
	**CC30**	776.21 ± 10.78 ^C^	6.62 ± 0.09 ^B^	1.50 ± 0.09 ^B^	23.11 ± 0.92
	**CC35**	871.77 ± 16.55 ^B^	7.14 ± 0.15 ^A^	1.51 ± 0.04 ^B^	23.26 ± 0.41
	**CC40**	922.32 ± 6.96 ^A^	7.09 ± 0.07 ^A^	1.68 ± 0.13 ^A^	25.73 ± 1.34

Values are expressed as mean of 3 replications (*n* = 3) ± standard deviation. The mean values with different letters (a, b, c, d, e, f or A, B, C, D, E, F) in the same column and within one series are statistically different (*p*-value < 0.05).

**Table 3:** The content of 5-hydroxymethylfurfural (HMF) and the browning index of wort produced with the use of different types and doses of special malts in the wort.

Series	Variant	Browning Index	HMF
		AU	mg/L
	**P**	0.62 ± 0.04 ^f,F^	0.66 ± 0.00 ^f,F^
**I**	**CJ**	1.80 ± 0.01 ^d^	19.65 ± 0.00 ^c^
	**CC**	4.43 ± 0.04 ^b^	20.90 ± 0.01 ^b^
	**PC**	3.16 ± 0.01 ^c^	5.54 ± 0.03 ^e^
	**JP**	5.57 ± 0.03 ^a^	24.31 ± 0.17 ^a^
	**JB**	1.05 ± 0.00 ^e^	12.32 ± 0.04 ^d^
**II**	**CC20**	10.05 ± 0.07 ^E^	51.81 ± 0.54 ^C^
	**CC25**	10.79 ± 0.16 ^D^	50.62 ± 0.14 ^D^
	**CC30**	13.58 ± 0.50 ^C^	47.62 ± 0.54 ^E^
	**CC35**	15.53 ± 0.39 ^B^	75.61 ± 0.43 ^B^
	**CC40**	17.99 ± 0.48 ^A^	91.94 ± 0.85 ^A^

Values are expressed as mean of 3 replications (*n* = 3) ± standard deviation. The mean values with different letters (a, b, c, d, e, f for A, B, C, D, E, F) in the same column and within one series are statistically different (*p*-value < 0.05).

**Table 4:** Analysis of the correlation between the antioxidant activity, total polyphenol content, color of the wort, 5-hydroxymethylfurfural (HMF) content and the carbohydrate profile of wort produced with 20–40% dark chocolate malt.

Variable	FRAP	ABTS^•+^	TPC	BI	HMF	Dextrin	Maltotriose	Maltose	Glucose
**FRAP**	1.00	0.83	0.91	0.94	0.79	0.87	−0.87	−0.86	0.32
**ABTS^•+^**		1.00	0.75	0.84	0.70	0.95	−0.62	−0.72	−0.01
**TPC**			1.00	0.93	0.87	0.82	−0.91	−0.91	0.17
**BI**				1.00	0.88	0.94	−0.88	−0.91	0.11
**HMF**					1.00	0.80	−0.88	−0.98	−0.17
**Dextrin**						1.00	−0.70	−0.81	−0.06
**Maltotriose**							1.00	0.94	−0.19
**Maltose**								1.00	0.04
**Glucose**									1.00

The marked correlation coefficients are significant with *p* < 0.05, *n* = 15.
